# Nutritional and Polyphenolic Composition of *Agrimonia procera* Wallr. from Experimental Cultivation with Different Levels of Nitrogen Fertilization

**DOI:** 10.3390/molecules27217597

**Published:** 2022-11-05

**Authors:** Elżbieta Karlińska, Olga Kaczorowska, Beata Romanowska, Monika Kosmala

**Affiliations:** 1 Institute of Food Technology and Analysis, Faculty of Biotechnology and Food Sciences, Lodz University of Technology, ul. Stefanowskiego 2/22, 90-537 Łódź, Poland; 2 Medicinal Plant Garden, Department of Pharmacognosy, Faculty of Pharmacy, Medical University of Lodz, Muszyńskiego 1, 90-151 Łódź, Poland

**Keywords:** fragrant agrimony, *Agrimonia procera* Wallr., nutrients, polyphenols, ellagitannins, HPLC-DAD-MS

## Abstract

Plants of the genus *Agrimonia*, including fragrant agrimony *Agrimonia procera* Wallr., mainly used as pharmaceutical raw material, perfectly fit into the current trends in nutrition and food technology that are searching for organic raw materials with high contents of bioactive compounds, such as dietary polyphenols and fiber. The aim of the research was to determine the nutritional and polyphenolic composition of fragrant agrimony *Agrimonia procera* Wallr. from experimental cultivation with varying levels of nitrogen fertilization in the nitrate, ammonium, or amide forms. In the experimental cultivation of fragrant agrimony in a seeding medium with a moderately high level of mineral content, the aerial parts, especially the leaves, were characterized by contents protein, dietary fiber, fat, and polyphenols at levels of 11.5, 58.0, 3.0, and 10.5% of dry matter, respectively, and an energy value of about 260 kcal/100 g of dry matter. The effect of additional nitrogen fertilization, in the form of ammonium nitrate and urea, on the content and yield of nutrients and polyphenol in fragrant agrimony depends both on the dose and the form of nitrogen, as well as the morphological part, of the plant.

## 1. Introduction

With the development of health beneficial-based foods and personalized nutrition, a demand for safe phytocompounds and herbal products with a positive effect on human health, such as reducing the risk of diet-related diseases, is increasing [1,2,3]. Such substances, in addition to dietary fiber, can include some groups of polyphenols, especially flavonoids (FV) and hydrolyzable tannins (ellagitannins, ET), found in fruits, vegetables, and herbs [1,4,5,6,7,8].

Flavonoids (FV) are most often divided into the following groups: flavones (FW), flavonols (FL), flavanones, flavan-3-ols, flavan-3,4-diols, isoflavonoids, anthocyanins, chalcones, and aurons. FV are usually in the form of O-glycosides and C-glycosides. Despite the fairly uniform structure, their pharmacological and therapeutic properties are significantly differentiated [6]. The rutoside (quercetin-3-O-rhamnoglucoside) FL increase the elasticity and permeability of blood vessels, while the quercetin-3-O-glucoside FL have a diuretic effect [9,10]. The luteolin FW have specific cytotoxicity in cancer cells, without damaging healthy ones (chemotherapeutic and anti-cancer properties) [8]. The apigenin FW have antibacterial anti-inflammatory effects, neuroprotectively accelerate the formation of nerve cells, and strengthen nerve connections in the brain, which can increase memory and learning functions [11].

ET are esters of 3,4,5,3′,4′,5′-hexahydroxydiphenyl acid (HHDP) and usually β-D-glucose (or its oligomers). These polyphenols are characterized by a high variability of their structure. ET and their metabolites exhibit antiviral, antibacterial, antioxidant, anti-inflammatory, and antimutagenic effects [4,5,12,13,14].

FV and tannins can be found in edible plants and herbs belonging to several genus of the family *Rosaceae*. A particularly rich source of FV and ET is the *Agrimonia* genus. In the Northern Hemisphere, there are more than 12 species of this genus, of which the most important, due to the properties and composition of active substances, are the following: *Agrimonia pilosa* Ledeb. (hairy agrimony), *Agrimonia eupatoria* L. (common agrimony), and *Agrimonia procera* Wallr. (synonym *Agrimonia odorata* Mill., fragrant agrimony). For centuries, traditional medicines of China and Japan have been using extracts of the *Agrimonia* root and herb as disinfectants and hemostatics [5,12,13]. According to the lexicon of medicinal plants, the dried herb of *Herba Agrimoniae*, including the upper shoots of plants of the genus *Agrimonia* from the species *A. eupatoria* L., *A. procera* Wallr., and *A. pilosa* Ledeb., is used as a pharmaceutical raw material (infusions, decoctions, and extracts) [15].

According to the current state of knowledge, *A. procera* Wallr. is a plant with high water and food requirements, but it is resistant to fungal diseases and rarely attacked by pests. So far, the plant has not been the subject of cultivation experiments with the use of nitrogen fertilization, which may affect the specific composition of agrimony polyphenols, such as secondary metabolites dependent on nitrogen assimilation and transformation of some amino acids.

The aim of the research was to expand the knowledge about the chemical composition of *A. procera* Wallr. from experimental cultivation with varying levels of nitrogen fertilization. The aim was also to identify the new perspectives for the use of fragrant agrimony as a rich source of phytocomponents, including dietary polyphenols and dietary fiber.

## 2. Results

Plants of the genus *Agrimonia L*., including fragrant agrimony *A. procera* Wallr., are characterized by a high yield of biomass—they are resistant to diseases and do not require the use of plant protection products. Moreover, the plants contain biologically active substances that affect human metabolism. *A. procera* Wallr., which, unlike common agrimony *A. eupatoria* L., does not contain substances with a strong cardiac effect, perfectly fits into the current trends in nutrition and food technology, which are searching for organic raw materials with a high content of bioactive natural compounds, such as polyphenols. It is, therefore, important to identify new practical perspectives for the use of fragrant agrimony in the food industry as a rich source of phytocomponents, including dietary polyphenols and dietary fiber. The data presented in Table 1, Table 2, Table 3 and Table 4 concerns the results of the determination of nutrients and polyphenols contained in a dried fragrant agrimony herb *A. procera* Wallr. with a dry matter content above 92%.

### 2.1. Dry Matter and Basic Nutrients in Selected Morphological Parts of A. procera Wallr.

The dry matter content in the leaves, stems, roots, and underground buds of fragrant agrimony grown in a sowing medium with a starting level of total nitrogen was 30.45, 35.73, 48.33, and 29.91 g/100 g, respectively (Table 1). Only in the case of the application of the first level of additional fertilization with ammonium nitrate was a tendency to increase the dry matter content in leaves and roots observed, compared to the other variants of the experiment.

The aboveground parts of fragrant agrimony, especially the leaves, were characterized by a high content of protein and dietary fiber, with a relatively low fat content. In the leaves of fragrant agrimony, the contents of protein and dietary fiber in plants grown in a seeding medium with a starting level of nitrogen fertilization were at levels of 11.5 and 58%, respectively (on average, in the whole experiment, 12% and 55%, respectively), while in the stems, they were 3.5 and 74%, respectively (on average, in the whole experiment, 7 and 75%, respectively).

The use of additional nitrogen fertilization had a statistically significant impact on the increase in protein content in all analyzed morphological parts of fragrant agrimony. The largest increase in protein content was achieved by using the second level of additional nitrogen fertilization in the form of ammonium nitrate in the case of leaves (protein content 16.77%) and, in the case of other analyzed morphological parts, using the second level of additional nitrogen fertilization in the form of urea (protein content in underground buds, stems, and roots, 16.65, 9.24, and 3.54%, respectively). The fertilizer variants used had little effect on the content of other nutrients (total ash and dietary fiber content in leaves and stems) or no effect at all (fat, total ash content in roots, and underground buds).

The energy values (calculated on the basis of the content of proteins, fats, dietary fiber, and carbohydrates, in accordance with the Commission Directive 2008/100/EC) of leaves and stems of fragrant agrimony were comparable, an average 260 and 240 kcal/100 g DM, respectively. The first level of additional nitrogen fertilization, in the form of ammonium nitrate, caused a significant increase in the energy value of the leaves, while the second level of additional nitrogen fertilization, in the form of ammonium nitrate, resulted in a significant reduction in the calorific value of fragrant agrimony stems, in relation to other variants of the experiment.

Nurzyńska et al. [16] showed that the dose of nitrogen significantly affected the chemical composition of basil herb, including protein content. Increasing the nitrogen dose from 0.2 to 0.9 g/dm^3^ resulted, depending on the variety, in an increase in protein content of 40–50%. In the research of Kotecki et al. [17], it was shown that, under the influence of increasing doses of nitrogen fertilizers (in the range of 60–150 kg/ha) in spring rapeseed, the raw fat content decreased (by 2%), and the protein content increased (by 5%).

### 2.2. Polyphenols in Selected Morphological Parts of A. procera Wallr.

The study investigated the effect of a dose and form of nitrogen on the content in leaves, stems, roots, and underground buds of the main groups of polyphenols, i.e., ET (a sum of agrimoniin, pedunculagin, and ellagic acid), flavan-3-ols (a sum of proanthocyanidins and free catechins), and FV, divided into FL (a sum of apigenin and luteolin glucuronides) and FW (a sum of quercetin and kaempferol glycosides). Table 2 presents data on the retention times, molecular weight (MW), and fragmentation ions (MS/MS spectrum) of the polyphenols identified in *A. procera* leaves, stems, roots, and underground buds. The ET identified in fragrant agrimony were agrimoniin and two isomers of pedunculagin (β-pedunculagin and α-pedunculagin). Ellagic acid, taking into account its chemical structure, belongs to the phenolic acids, but considering the fact that its non-lactonized form (hexahydroxydiphenic acid) is the basic building unit, in this study, it was included in the ET group. The main flavones identified in *A. procera* Wallr. were glucuronides of apigenin and luteolin, while among flavonols, the presence of quercetin 3-*O*-rhamnoglucoside, quercetin 3-*O*-rhamnoglucoside, quercetin 3-*O*-galactoside, kaempferol 3-*O*-glucoside, two isomers of kaempferol-3-O-β-d-(6″-E-p-coumaroyl)-glucopyranoside (KpCG), and a compound tentatively identified as quercetin arabinoglycoside were identified. NMR analysis is required to determine the type of sugar substituent (glucose or galactose) in the quercetin arabinoglycoside molecule. The quantitative analysis of flavan-3-ols was conducted with the fluoroglucinolysis method, which allows for the determination of the total content of this group of compounds. Therefore, Table 2 shows the identification of selected major compounds belonging to flavan-3-ols (dimeric, trimeric, tetrameric procyanidins, catechin, and epicatechin). ET, ellagic acid, and flavan-3-ols were found in all analyzed parts of fragrant agrimony (leaves, stems, and underground buds). In contrast, FL and FW were found only in the analysed aboveground parts of the plant (leaves, stems, and underground bud). Derivatives of FL and FW have not been identified in the roots of fragrant agrimony. Our findings are in agreement with the results of other research studies concerning the polyphenolic profile of *Agrimonia* spp. [18,19,20,21,22,23].

**Table 2:** LC-MS identification of polyphenols in leaves, stems, fruits, roots, and underground buds of *Agrimonia procera* Wallr.

Peak No.	Compound	RT [min]	UV [nm]	MS Data [*m*/*z*]	MS/MS Data	Occurrence		Identification
						*A. procera* Wallr.		
						L S Ub	R	
1	β-pedunculagin	11.8	244 sh	[783.07]^−1^	613, 481, **301**	+	+	Standard
2	α-pedunculagin	17.9	248 sh	[783.07]^−1^	613, 481, **301**	+	+	Standard
3	Procyanidin dimer	20.2	282	[577.14]^−1^	451, 425, 407, 289	+	+	[18]
4	Procyanidin trimer	20.7	282	[865.20]^−1^	695, 577, 451, 425, 289	+	+	[18]
5	Epicatechin	21.6	289	[289.07]^−1^	271, 245, 205, 179	+	+	[18]
6	Catechin	22.5	281	[289.07]^−1^	271, 245, 205, 179	+	+	[18]
7	Procyanidin tetramer	23.4	283	[1153.26]^−1^	865, 695, 575, 449, 287	+	+	
8	Quercetin arabinoglycoside ^a^	35.5	257, 355	[595.14]^−1^	463, 445, **301** *	+	-	[22,23]
9	Agrimoniin	36.3	260 sh	[934.08]^−2^	1567, 1235, 1085, 935, 897, 783, 633, **301**	+	+	Standard, [18,19,22]
10	Quercetin 3*-O-*rhamnoglucoside	38.3	257, 354	[609.12]^−1^	463, 343, **301**	+	-	Standard, [18,19,22]
11	Ellagic acid	38.8	254, 350	[301.10]^−1^	-	+	+	Standard, [18,22]
12	Quercetin 3*-O-*galactoside	39.4	257, 353	[463.08]^−1^	343, **301**	+	-	Standard, [19,21,22,24]
13	Kaempferol 3*-O-*glucoside	40.0	267, 350	[447.09]^−1^	327, **285**, 269, 255, 151	+	-	Standard, [19,20,21,22]
14	Luteolin 7*-O-*glucuronide	40.6	266, 349	[461.07]^−1^	357, 327, **285**, 175, 151, 113	+	-	Standard, [18,20,21,22,24]
15	Apigenin 7*-O-*glucuronide	43.1	268, 339	[445.07]^−1^	**269**, 175, 113	+	-	Standard, [18,20,21]
16	KpCG *	46.2	269, 315	[593.12]^−1^	447, 307, **285**	+	-	Standard, [18,23,24]
17	KpCG * isomer	46.7	269, 315	[593.12]^−1^	447, 307, **285**	+	-	[18,23,24]

RT—retention time; a—tentative assignment based on MS and UV–Vis spectra and literature data; MS data and MS/MS data are experimental values; MS/MS ions marked in bold font represent fragments with intensity >50%, relative to the maximum intensity; standard—identification based on the standard compound; L—leaf, S—stem, Ub—underground buds, R—roots; KpCG *—kaempferol-3-O-β-d-(6″-E-p-coumaroyl)-glucopyranoside; sh—shoulder.

The contents of individual polyphenols in fragrant agrimony are given in Table S1 in the Supplementary Materials, while Table 3 shows the contents of polyphenols, with the division into the main groups of these compounds. According to the data presented in Table 3, ET and flavan-3-ols are the dominant polyphenols of all considered morphological parts of fragrant agrimony. In plants grown in a seeding medium with a starting level of nitrogen fertilization, the ET content, calculated on dry matter, was almost 5% in leaves and stems, 8% in roots, and 16% in underground buds, with the agrimoniin accounting for about 98% of the determined sum of ET (Table S1). Flavan-3-ols, calculated on dry matter, were present in fragrant agrimony in amounts of almost 3.7% in leaves, over 2% in stems and underground buds, and 4.6% in roots. FL and FW in fragrant agrimony were present in total amounts not exceeding 2% of the dry mass of leaves, 1.5% of the mass of underground buds, and slightly more than 0.1% of the mass of stems.

**Table 3:** The content of main polyphenol groups (g/100 g DM) in various morphological parts of fragrant agrimony *Agrimonia procera* Wallr. From experimental cultivation.

Morphological Part of Plant	Seed Bed with a Nitrogen Content of 140 mg/dm^3^			
	Additional Nitrogen Dose And Type Of Fertilizer			
	0	25 mg/dm^3^ Ammonium Nitrate	100 mg/dm^3^ Ammonium Nitrate	100 mg/dm^3^ Urea
Ellagitannins *				
Leaves	4.90 ± 0.41 ^a,b^	4.95 ± 0.45 ^a,b^	3.94 ± 0.26 ^a,b^	3.78 ± 0.41 ^a^
Stems	4.80 ± 0.17 ^a,b^	5.80 ± 0.40 ^b^	5.25 ± 0.5 ^a,b^	5.31 ± 0.9 ^a,b^
Roots	8.02 ± 1.00 ^c^	8.44 ± 0.60 ^c^	7.87 ± 0.94 ^c^	8.29 ± 0.63 ^c^
Underground buds	15.52 ± 2.17 ^f^	11.01 ± 2.93 ^d^	10.15 ± 1.55 ^d^	13.76 ± 5.04 ^e^
Flavan-3-ols **				
Leaves	3.67 ± 0.44 ^c,d,e^	3.31 ± 0.24 ^c,d^	3.86 ± 0.40 ^d,e^	3.98 ± 0.31 ^e^
Stems	2.33 ± 0.2 ^a,b^	1.98 ± 0.25 ^a,b^	2.51 ± 0.36 ^b^	3.19 ± 0.37 ^c^
Roots	4.62 ± 0.35 ^f^	3.76 ± 0.27 ^c,d,e^	3.91 ± 0.28 ^e^	3.24 ± 0.36 ^c^
Underground buds	2.52 ± 0.24 ^b^	1.88 ± 0.11 ^a^	2.09 ± 0.27 ^a,b^	2.27 ± 0.03 ^a,b^
Flavonols ***				
Leaves	0.98 ± 0.08 ^c^	0.77 ± 0.06 ^b^	0.76 ± 0.03 ^b^	0.74 ± 0.03 ^b^
Stems	0.12 ± 0.01 ^a^	0.11 ± 0.01 ^b^	0.09 ± 0.01 ^a^	0.08 ± 0.01 ^a^
Roots	nd	nd	nd	nd
Underground buds	0.97 ± 0.26 ^c^	0.78 ± 0.12 ^b^	0.84 ± 0.22 ^b^	0.82 ± 0.21 ^b^
Flavons ****				
Leaves	0.94 ± 0.03 ^f^	0.80 ± 0.02 ^e^	0.73 ± 0.05 ^d^	0.72 ± 0.03 ^d^
Stems	0.01 ± 0.0 ^a^	0.02 ± 0.0 ^a^	0.01 ± 0.00 ^a^	0.02 ± 0.01 ^a^
Roots	nd	nd	nd	nd
Underground buds	0.46 ± 0.13 ^c^	0.39 ± 0.07 ^b^	0.38 ± 0.11 ^b^	0.42 ± 0.14 ^b,c^
Sum of polyphenols				
Leaves	10.50 ± 0.2 ^d,e^	9.83 ± 0.57 ^c,d^	9.29 ± 0.33 ^b,c,d^	9.22 ± 0.39 ^b,c,d^
Stems	7.34 ± 0.31 ^a^	7.91 ± 0.39 ^d,e^	7.87 ± 0.66 ^a,b^	8.60 ± 0.77 ^a,b,c^
Roots	12.59 ± 1.17 ^f^	12.31 ± 0.55 ^f^	11.79 ± 0.96 ^e,f^	11.55 ± 0.82 ^e,f^
Underground buds	19.86 ± 0.8 ^h^	14.90 ± 2.8 ^g^	12.99 ± 1.9 ^f^	18.94 ± 4.4 ^h^

Mean value ± standard deviation (*n* = 4); DM—dry matter; ellagitannins *—sum of agrimoniin, pedunculagin, and ellagic acid; flavan-3-ols **—sum of proanthocyanidins and free catechins; flavons ***—sum of apigenin and luteolin glucuronides; flavonols ****—sum of quercetin and kaempferol glycosides; nd—not detected; a–h—values relating to the content of main groups of polyphenols, depending on the morphological part of the plant, and the additional nitrogen dose and type of fertilizer denoted by the same letter do not differ statistically significantly at *p* ≤ 0.05.

Karlińska et al. [18] showed that the agrimoniin content in three-year-old fragrant agrimony, derived from a field crop, in which, apart from compost sown in spring, no additional nitrogen fertilization was used, was lower and amounted to 2 and 3% of dry matter in the leaves and stems during the flowering phase, respectively. According to Granica et al. [20], the agrimoniin content of fragrant agrimony herb was in the range of 21–37 mg/g DM. Gräber et al. [25] showed that powdered leaves and powdered upper shoots of fragrant agrimony contain 12.7 and 6.4 mg of agrimoniin in 1 g of dry matter, respectively.

Common agrimony is characterized by a much lower content of agrimoniin, compared to fragrant agrimony. Granica et al. [20] showed that the content of agrimoniin in six samples of common agrimony herb was in the range of 1.2–6.8 mg/g DM, while Karlińska et al. [18] showed that the leaves and stems of common agrimony contain agrimoniin in the amount of 4.2 and 2.3 mg/g DM, respectively.

The effect of additional nitrogen fertilization on the content of the main groups of polyphenols in agrimony depended on both the dose and form of nitrogen, as well as the morphological part of the plant. In the case of increasing the additional nitrogen fertilization by 25 mg/dm^3^ in the form of ammonium nitrate, the ET content in the leaves did not change, while in the stems, it increased significantly, compared to plants grown in a substrate with a starting level of fertilization. On the other hand, a further increase in nitrogen fertilization by 100 mg N/dm^3^ in both ammonium nitrate and the urea resulted in a significant reduction in the ET content in the leaves, while in the stems, there was only a tendency to reduce the content of this group of polyphenols under the influence of the increased dose of nitrogen.

The specific effect of nitrogen in the amide form was visible, in relation to the content of flavan-3-ols in agrimony. Urea significantly increased the content of flavan-3-ols in the stems, while reducing their content in the roots. The nitrate and ammonium form of nitrogen, introduced with an additional dose of ammonium nitrate, did not affect the content of flavan-3-ols in the stems, while in the roots, it caused a decrease in the content of this group of polyphenols, compared to plants grown in a substrate with a starting level of fertilization.

The applied variants of additional fertilization resulted in a significant reduction in the total content of polyphenols in agrimony leaves, and in the stems, only urea significantly increased the content of the sum of polyphenols, while the first and second levels of additional fertilization with ammonium nitrate reduced the content of polyphenols in underground buds, compared to plants grown in a substrate with a starting level of NPK fertilization and plants additionally fertilized with urea.

The results of Kazimierczak et al. [26] study indicate a higher content of FV and phenolic acids in lemon balm, mint, lovage, and thyme from organic cultivation using organic fertilization in the form of manure, compared to the same herbs from conventional cultivation using mineral fertilization. The authors obtained the inverse effect only in the case of sage. Mudau et al. [27] showed that addition of nitrogenous fertilizer supplements (applied as limestone ammonium nitrate) resulted in significantly increased concentrations of total polyphenols in *Athrixia phylicoides* L. leaves, with the most prominent increases evident with nitrogenous applications in the range 0 to 100 kg N/ha; however, a further increase in the nitrogen dose to 500 kg N/ha did not cause a significant increase in the polyphenol content in bush tea.

### 2.3. Yield of Selected Nutrients and Polyphenols in A. procera Wallr.

According to the data presented in Table 4, the use of additional nitrogen fertilization in the form of ammonium nitrate or urea significantly increased the protein yield in the stems, leaves, roots, and underground buds of agrimony and the yield of dietary fiber and fat in the leaves and stems of the plant, compared to plants grown on a substrate with a starting level of nitrogen fertilization. The yield-generating effect of additional nitrogen fertilization was particularly visible for protein.

**Table 4:** Yield of dry matter, selected nutrients, and main groups of polyphenols in various morphological parts of fragrant agrimony *Agrimonia procera* Wallr. from experimental cultivation.

Morphological Part of Plant	Seed Bed with a Nitrogen Content of 140 mg/dm^3^			
	Additional Nitrogen Dose and Type of Fertilizer			
	0	25 mg/dm^3^ Ammonium Nitrate	100 mg/dm^3^ Ammonium Nitrate	100 mg/dm^3^ Urea
Average amount of fresh mass (g)				
Whole plant	33.91 ± 6.36	38.24 ± 8.80	40.13 ± 7.17	34.30 ± 4.91
Average amount of dry matter (g)				
Whole plant	12.28 ± 2.91	14.90 ± 3.26	14.07 ± 2.88	11.79 ± 0.98
Protein (mg)				
Leaves	498 ± 7 ^i^	943 ± 31 ^l^	916 ± 7 ^k^	714 ± 10 ^j^
Stems	76 ± 3 ^a^	188 ± 1 ^e^	257 ± 4 ^g^	216 ± 7 ^f^
Roots	83 ± 2 ^a^	114 ± 2 ^b,c^	123 ± 2 ^c,d^	95 ± 2 ^a,b^
Underground buds	140 ± 2 ^d^	257 ± 7 ^g^	286 ± 4 ^h^	261 ± 1 ^g^
Dietary fiber (mg)				
Leaves	2510 ± 24 ^e^	3113 ± 199 ^f^	3133 ± 10 ^f^	3093 ± 22 ^f^
Stems	1543 ± 6 ^a^	1966 ± 17 ^c^	2306 ± 49 ^d^	1745 ± 10 ^b^
Fat (mg)				
Leaves	117 ± 4 ^b^	163 ± 7 ^e^	147 ± 8 ^d^	137 ± 1 ^c^
Stems	10 ± 1 ^a^	12 ± 1 ^a^	13 ± 1 ^a^	10 ± 1 ^a^
Ellagitannins * (mg)				
Leaves	211 ± 30 ^b^	298 ± 66 ^c^	208 ± 16 ^b^	194 ± 17 ^b^
Stems	101 ± 22 ^a^	160 ± 54 ^a,b^	157 ± 51 ^a,b^	109 ± 7 ^a^
Roots	361 ± 104 ^c^	368 ± 109 ^c^	300 ± 107 ^c^	221 ± 57 ^b^
Underground buds	209 ± 60 ^b^	213 ± 91 ^b^	188 ± 26 ^b^	197 ± 93 ^b^
Agrimoniin (mg)				
Leaves	207 ± 29 ^b,c^	292 ± 65 ^c,d,e^	204 ± 15 ^b,c^	191 ± 17 ^b,c^
Stems	99 ± 21 ^a^	156 ± 52 ^a,b^	153 ± 51 ^a,b^	107 ± 7 ^a^
Roots	357 ± 104 ^e^	365 ± 109 ^e^	297 ± 106 ^d,e^	218 ± 57 ^b,c,d,e^
Underground buds	208 ± 60 ^b,c^	213 ± 91 ^b,c,d^	188 ± 26 ^a,b,c^	212 ± 52 ^b,c,d^

Mean value ± standard deviation; DM—dry matter; ellagitannins *—sum of agrimoniin, pedunculagin, and ellagic acid; a–l—values relating to to the yield of dry matter, selected nutrients, and main groups of polyphenols, depending on the morphological part of the plant, and the additional nitrogen dose and type of fertilizer, denoted by the same letter, do not differ statistically significantly at *p* ≤ 0.05.

The fertilizer combinations used resulted in an almost three-fold increase in protein yield in the stems and two-fold increase in the underground buds. After the application of ammonium nitrate (first and second level of fertilization), the protein yield, compared to plants grown in a substrate with a starting level of nitrogen fertilization in the leaves, increased almost 2-fold, in the roots 1.5-fold, and after the application of urea the effect was lower, and the protein yield increased in the leaves by 1.5-fold and in the roots by 1.1-fold. It is worth noting that increasing the dose of nitrogen in the form of ammonium nitrate from 25 to 100 mg N/dm^3^ did not cause an increase in the yield of nutrients and polyphenolic in agrimony, proportional to the dose of nitrogen (protein yield in leaves, roots, and underground buds), and even in the case of some components it resulted in a decrease in their yield (ET and agrymoniin). A similar effect of increasing the yield of fat and protein under the influence of applied nitrogen fertilization was obtained by Kotecki et al. [17] in the case of spring rape. When fertilization increased from 60 to 150 kg N/ha, the efficiency of raw fat extraction increased by 19%, and total proteins increased by 25%.

With regard to polyphenols, the yield-forming effect of nitrogen was much weaker than in the case of nutrients and was manifested only by an increased yield of ET and agrimoniin in the leaves and stems of fragrant agrimony under the influence of additional fertilization with ammonium nitrate at a dose equivalent to 25 mg N/dm^3^, compared to plants grown on a substrate with a starting level of nitrogen fertilization (the increase in ET and agrimoniin yield by 50%, on average). With the exception of the stems, increasing the dose of nitrogen introduced with ammonium nitrate to 100 mg N/dm^3^ resulted in a decrease in ET and agrimoniin yield in the leaves, roots, and underground buds by 30, 20, and 10%, respectively.

Urea at a dose equivalent to 100 mg N/dm^3^ had no effect on the amount of ET and agrimoniin yield in the leaves, stems, and underground buds, while in the roots, it caused a significant reduction in the yield of these polyphenols, in comparison to plants grown in a substrate with a starting nitrogen fertilization level.

The presented data indicate the prospect of using fragrant agrimony as a nutritional raw material. Especially the leaves and stems of this plant, which are high in dietary fiber, protein, and polyphenols, in raw form or after culinary processing, could be used as an ingredient in fruit and vegetable preparations.

## 3. Materials and Methods

### 3.1. Plant Material

Plant material (seeds) for research were obtained from the Medicinal Plants Garden (MPG) of the Department of Pharmacognosy, Faculty of Pharmacy, Medical University of Lodz, Łódź, Poland). The authenticity of the plant material was confirmed by the MPG, based on the morphological features of *Agrimonia procera* Wallr., using literature sources [28,29]. The voucher specimen 20180613/ARPR/MPG for *A. procera* Wallr. was deposited in MPG.

### 3.2. Seeding Medium

UAB Durpeta Sepeta seeding medium (Sphagnum moss peat with pH 5.5–6.5; organic matter content 92–96%), with the addition of calcium meal and fertilizer containing nitrogen, phosphorus, and potassium (NPK) with microelements and a water-absorbing substance. The substrate has a porous structure and is intended for sowing and transplanting seedlings. Bulk weight 250 g/dm^3^, humidity 65%. Chemical composition in (mg/dm^3^) was as follows: 125 N-NO_3_; 15 N-NH_4_; 27 P; 100 K; 64 Mg; 0.24 B; 1.4 Cu; 1.7 Zn; 67 Fe; 3.3 Mn; 8.0 Na.

### 3.3. Nitrogen Fertilizers

Ammonium nitrate (NH_4_NO_3_; 34% N) and urea (CO(NH_2_)_2_; 46% N). Aqueous stock solutions with a substance content of 10 mg/mL have been prepared from each fertilizer and, in the right proportion with water, were used for soil fertilization of plants.

### 3.4. Conduct of the Experiment

Before sowing, achenes were stratified in a mixture of moist seedbed and sand (in a ratio 1:1), in the proportion of seeds-to-substrate 1:10, at 1–5 °C for 5 weeks. The sprouted seeds were sown into boxes with the seeding medium, 0.5–1 cm deep, with a spacing of 5 × 8 cm. The boxes with seeds were covered with glass, placed in a foil tunnel, and protected against frost by covering with a mat. Occasionally ventilated and irrigated. The fragrant agrimony seedlings were used for pot cultivation, in order to evaluate the effect of nitrogen dose and source on nutrient accumulation and polyphenol content in the plant, until the flowering phase, when the aerial parts were harvested as herbal raw material. The object of the comparison was four variants of agrimony growing: (1) in the sowing substrate with NPK content, 140, 27, 100 mg/dm^3^, (2) in the sowing substrate enriched with mineral nitrogen in the amount of 25 mg N/dm^3^ (1st level of additional fertilization) in the form of ammonium nitrate (Σ 165 mg N/dm^3^, including 137.5 mg/dm^3^ N-NO_3_; 27.5 mg/dm^3^ N-NH_4_), (3) in the sowing medium enriched with mineral nitrogen in the amount of 100 mg N/dm^3^ (2nd level of additional fertilization) in the form of ammonium nitrate (Σ 240 mg N/dm^3^, including 175 mg/dm^3^ N-NO_3_; 65 mg/dm^3^ N-NH_4_), (4) in the sowing medium enriched with organic nitrogen in the amount of 100 mg/dm^3^ (2nd level of additional fertilization) in the form of urea (Σ 240 mg N/dm^3^, including 125 mg/dm^3^ N-NO_3_; 15 mg/dm^3^ N-NH_4_; 100 mg/dm^3^ N-NH_2_). Each variant of the experiment consisted of two pots (containing four plants).

The experimental cultivation was performed with the use of eight vases with a capacity of 3 dm^3^ (diameter 14 cm, height 21 cm), which were completely filled with the fresh, above-mentioned substrate. Two fragrant agrimony seedlings in the phase of three true leaves were planted in each of the eight pots, and they were thoroughly irrigated by adding 350 mL of tap water to the surface of each pot. The plants in pots were placed at the temperature of 18–23 °C in a foil tunnel for adaptive vegetation for 14 days. During this period, the plants were occasionally watered with a small amount of settled tap water (approx. 100 mL/pot) to compensate for the losses by evaporation. After 14 days of adaptive vegetation, additional fertilization was started with the use of ammonium nitrate and urea, respectively. The next three soil nitrogen fertilization treatments were performed during the cultivation, at intervals of three weeks. The calculated amounts of nitrogen demand were applied to the soil in four equal doses, respectively, 55 mg (1st level) and 220 mg (2nd level) of ammonium nitrate and 163 mg (2nd level) of urea fertilization. Each time, the measured amount of ammonium nitrate and urea stock solution was added to 400 mL of settled tap water and, after thorough mixing, was evenly poured on the surfaces of vases with plants. In the case of growing fragrant agrimony in a sowing medium without additional nitrogen fertilization, the plant was watered with 400 mL of settled tap water. The experiment ended when the plants were in the initial flowering phase. At that time, the plants were removed from individual vases, the substrate was separated from the roots by gentle shaking. The yield of fresh weight of each plant was determined. In order to determine the structure of the crop, each plant was separated into the main morphological parts, i.e., roots, leaves, stems, and underground buds (young shoots), and weighed. The individual morphological parts of the plants were dried in a convection dryer (POL-Eko, Poland), at a temperature of 35 °C for 62 h, and then crushed in the laboratory mill IKA A11 (IKA-Analytical Mill, Staufen, Germany). The determination of the polyphenol content of roots, leaves, stems, and underground buds was made separately for each of the four plants included in the experimental variant. However, in order to determine the basic nutrients, the relevant morphological parts of the plants included in a given variant of the experiment were combined.

### 3.5. Determination of the Nutritional Composition of Fragrant Agrimony

The determination of the nutritional composition was performed in accordance with the AOAC official method, respectively: dry substance and total ash—AOAC official method 940.26 [30]; proteins by the Kjeldahl method—AOAC official method 920.152 [31]; fat by the Soxhlet method using petroleum ether—AOAC official method 930.09 [32]; dietary fiber by enzymatic-weight method—AOAC official method 985.29 [33]. All determinations were made in two repetitions, the results were presented in g/100 g of dry matter.

### 3.6. Determination of Ellagitannins, Flavonols, and Flavons

#### 3.6.1. Extraction of Ellagitannins, Flavolols, Flavons from Agrimonia Procera Leaves, Stems, Roots, and Underground Buds

Extraction of ET, FW, and FL from *A. procera* stems and roots was performed three times in a mixture of acetone/water/formic acid (70/29.9/0.1 *v*/*v*/*v*) (mixture A), according to the method described by Karlińska et al. [18]. Approximately 350 mg of ground material was weighed into 7 mL polypropylene test tubes with stoppers, and 4 mL of mixture A was added, mixed thoroughly using a vortex, and placed in an IS-4 ultrasonic bath (Intersonic, Olsztyn, Poland) for 15 min. After that time, the contents of the tubes were centrifuged in an MPW-260R laboratory centrifuge (Med Instruments, Warsaw, Poland) at 10,000× *g*. Subsequently, the supernatant was decanted from the pellet into 10 mL volumetric flasks. The residue remaining in the tube was subjected to two further extractions, with 3 mL of mixture A, in the same way. The resulting extracts were combined in a 10 mL volumetric flask and filled to the mark with mixture A. Extracts obtained with mixture A were diluted in methanol in a 1:5 (*v*/*v*) ratio, centrifuged in a MPW-260R laboratory centrifuge (Med Instruments, Warsaw, Poland) at 16,000× *g*, transferred to chromatographic vials, and subjected to HPLC analysis, as described in Section 3.6.2 and Section 3.6.3. Extraction was performed in duplicate for each sample.

Extraction of polyphenols from *A. procera* leaves and underground buds was performed successively: three times in a 70/29.9/0.1 (*v*/*v*/*v*) mixture of methanol/water/formic acid (mixture B), and then three times in mixture A, in accordance with the methodology described by Karlińska et al. [18]. Extraction of polyphenols from leaves and underground buds was performed as described for stems and roots, except that the residue from extraction with mixture B was subjected to an analogous extraction with mixture A. The extracts obtained with mixtures B and A were collected separately into two 10 mL volumetric flasks. The extracts obtained with mixture B were diluted in methanol in a 1:4 (*v*/*v*) ratio, while those obtained with mixture A were diluted in methanol in a 1:2 (*v*/*v*) ratio. Subsequently, the diluted extracts were centrifuged in an MPW-260R laboratory centrifuge (Med Instruments, Warsaw, Poland) at 16,000× *g*, transferred to chromatographic vials, and subjected to HPLC analysis, as described in Section 3.6.2 and Section 3.6.3. Extraction was performed in duplicate for each sample.

#### 3.6.2. Identification of Polyphenols

The main polyphenols of *A. procera* were identified exactly according to the methodology described in a previous publication (Karlińska et al. [18]), using a Dionex Ultimate 3000 HPLC, coupled with a DAD and Q Exactive Orbitrap mass spectrometer (MS) (Thermo Fisher Scientific, Waltham, MA, USA). The separation was carried out on 250 mm×4.6 mm i.d., 5μm, Luna C18(2) 100 Å column (Phenomenex, Torrance, CA, USA) by gradient elution with solvent A—1% (*v*/*v*) formic acid in water and solvent B—63:20:17 (*v*/*v*/*v*) mixture of acetonitrile/methanol/water. The column temperature—35 °C; the flow rate—1 mL/min; the injection volume—20 μL. The following gradient was used: 0–6 min, 5% (*v*/*v*) B; 6–36 min, 5–28% (*v*/*v*) B; 36–48 min, 28–73% (*v*/*v*) B; 48–54 min, 73% (*v*/*v*) B; 54–60 min, 73–5% (*v*/*v*) B; 60–70 min, 5% (*v*/*v*) B. Data were collected using Xcalibur software (Thermo Fisher Scientific, Waltham, MA, USA). The MS system coupled to the HPLC was an Orbitrap mass spectrometer equipped with an H-ESI probe used in the negative mode. The source parameters were as follows: vaporizer temperature—500 °C, ion spray voltage—4 kV, capillary temperature—400 °C; and sheath gas and auxiliary gas flow rates—75 and 20 units, respectively. The detector was operated in either the full MS or full MS/dd-MS^2^ scan modes. In the full MS mode, the scan rage of *m*/*z* 200−2000 was used. To generate MS^2^ data, we used the full MS/dd-MS^2^ scan mode. The results of polyphenols identification are given in Table 2.

#### 3.6.3. Quantitation of Polyphenols

The contents of ET, FW, and FL were determined in accordance with the methodology described in the previous publication (Karlińska et al. [18]) using a Smartline (Knauer, Berlin, Germany) chromatograph coupled with a PDA detector. The separation was carried out on a 250 × 4.6 mm, i.d. 5 μm, Gemini C18 110 Å column (Phenomenex, Torrance, CA, USA) by gradient elution with solvent A—0.05% (*v*/*v*) phosphoric acid in water and solvent B—0.05% phosphoric acid in a 63:20:17 (*v*/*v*/*v*) mixture of acetonitrile/methanol/water. The column temperature—35 °C; the flow rate—1.25 mL/min; the injection volume—20 μL. The following gradient was used: 0–5 min, 5% (*v*/*v*) B; 5–30 min, 5–28% (*v*/*v*) B; 30–40 min, 28–73% (*v*/*v*) B; 40–45 min, 73% (*v*/*v*) B; 45–47 min, 73–5% (*v*/*v*) B; and 47–56 min, 5% (*v*/*v*) B. Data were collected using ClarityChrom v. 3.0.5.505 software (Knauer, Berlin, Germany). Agrimoniin and pedunculagin was detected at 250 nm, while FW, FL, and ellagic acid were detected at 360 nm. Calculation of the polyphenol content was performed on the basis of calibration curves prepared for individual standards. Table S2 in the Supplementary Materials contains validation parameters. The method of isolating the agrimoniin and pedunculagin standard was described in the previous publication [24]. The remaining chromatography standards were purchased from Extrasynthese (Genay, France). Agrimoniin, pedunculagin, ellagic acid, quercetin 3*-*O*-*rhamnoglucoside, quercetin 3*-O-*galactoside, kaempferol 3*-O-*glucoside, and kaempferol-3-O-β-d-(6″-E-pcoumaroyl)-glucopyranoside were quantified using their respective standards. Quercetin arabinoglycoside was quantified as quercetin 3*-O-*glucoside equivalents; luteolin 7*-O-*glucuronide was quantified as luteolin equivalents, and apigenin 7*-O-*glucuronide was quantified as apigenin 7-O-glucoside. The determination of the polyphenol content of roots, leaves, stems, and underground buds was performed in duplicate separately for each of the four plants included in the experimental variant. The sum of α-pedunculagin, β-pedunculagin, agrimoniin, and ellagic acid was expressed as ET content. The sum of quercetin arabinoglycoside, quercetin 3*-O-*rhamnoglucoside, quercetin 3*-O-*galactoside, kaempferol 3*-O-*glucoside, and kaempferol-3-O-β-d-(6″-E-pcoumaroyl)-glucopyranoside (tiliroside) was expressed as FW content. The sum of apigenin 7-O-glucuronide and luteolin 7-O-glucuronide was expressed as FL content. All results were expressed as mean value in mg/100 g DW and presented in Table 3 (the content of the main groups of polyphenols, i.e., ET, flawan-3-ols, FW, and FL) and Table S1 in Supplementary Materials (the content of the individual polyphenolic compound).

### 3.7. Determination of Flavan-3-ols (A Sum of Proanthocyanidins and Catechins)

The determination of flavan-3-ols content was performed by the method of degradation of proanthocyanidins in an acidic medium using an excess of phloroglucinol, according to Milala et al. [34]. The products of acid degradation of polymeric proanthocyanidins and free catechins were separated by the use of Knauer Smartline chromatograph (Berlin, Germany), equipped with UV–Vis detector (PDA 280, Knauer, Berlin, Germany) and fluorescent detector (FD; Shimadzu RF-10Axl, Kyoto, Japan). The separation was carried out on a 250 mm × 4.6 mm i.d., 5 μm, Gemini C18 110 Å column (Phenomenex, Torrance, CA, USA) by gradient elution with solvent A—2,5% (*v*/*v*) acetic acid in water—and solvent B—80% (*v*/*v*) acetonitrile in water. The column temperature—30 °C; the flow rate—1 mL/min; the injection volume—20 μL. The following gradient was used: 0–10 min, 4–7% (*v*/*v*) B; 10–27 min, 7–30% (*v*/*v*) B; 27–30 min, 30–70% (*v*/*v*) B; 30–34 min, 70% (*v*/*v*) B; 34–35 min, 70–4% (*v*/*v*) B; 35–50 min, 4% (*v*/*v*) B. The content of flawan-3-ols is presented in Table 3 and in Table S1 in Supplementary Materials.

### 3.8. Statistical Analysis

The results are expressed as means and pooled SEM. Statistical analysis was conducted using Statistica 12 software (StatSoft, Tulsa, OK, USA). Statistical analysis included the effect of morphological part of the plant and the additional dose and form of nitrogen fertilization on the content and yield of individual nutrients and polyphenols in fragrant agrimony. Two-way analysis of variance (ANOVA) was used to evaluate the effects of morphological part of the plant (MP, leaves, stems, roots, and underground buds), additional nitrogen dose and type of fertilizer (NF, 0 mg/dm^3^; 25 mg/dm^3^ ammonium nitrate; 100 mg/dm^3^ ammonium nitrate; 100 mg/dm^3^ urea), and interactions between these two factors (MP × NF). If the analysis revealed a significant interaction (*p* ≤ 0.05), differences between the respective study groups were determined with Duncan’s post hoc test at *p* ≤ 0.05.

## 4. Conclusions

The results of the conducted research indicate that fragrant agrimony, so far used only as a pharmaceutical raw material, is a valuable raw material with potential food use, and it perfectly fits into the current trends in nutrition and food technology. The upper shoots of fragrant agrimony, and especially the leaves, are a source of nutrients and dietary polyphenols (with the content of protein, dietary fiber, fat, and polyphenols at the levels of 11.5%; 58.0%, 3%, and 10.5% of dry matter, respectively). The main polyphenols of fragrant agrimony were ET and flavan-3-ols (with contents in the leaves of 4.90% and 3.70% of dry matter, respectively). Agrimoniin accounted for about 98% of the determined sum of ET and was present in all analyzed parts of the plant. The yield-forming effect of additional nitrogen fertilization associated with the fertilizer combinations used resulted in almost a three-fold increase in protein yield in the stems and two-fold in leaves and underground buds, as well as a 50% increase in the yield of total dietary fiber and ET (mostly agrimoniin) in the leaves and stems of fragrant agrimony.

The obtained research results indicate that the analyzed raw material is a valuable source of health-promoting ingredients and has been shown to be suitable not only for pharmacological purposes, but also as a food additive.

## Figures and Tables

**Table 1:** The dry matter, nutrients, and energy value of selected morphological parts of fragrant agrimony *Agrimonia procera* Wallr. from experimental cultivation.

Morphological Part of Plant	Seed Bed with a Nitrogen Content of 140 mg/dm^3^			
	Additional Nitrogen Dose and Type of Fertilizer			
	0	25 mg/dm^3^ Ammonium Nitrate	100 mg/dm^3^ Ammonium Nitrate	100 mg/dm^3^ Urea
Dry matter (g/100 g)				
Leaves	30.45 ± 2.37 ^a,b^	34.45 ± 1.14 ^a,b,c^	31.81 ± 0.76 ^a,b,c^	31.03 ± 1.71 ^a,b,c^
Stems	35.73 ± 1.99 ^a,b,c,d^	36.21 ± 1.48 ^c,d^	37.01 ± 0.41 ^c,d^	34.50 ± 0.76 ^a,b,c^
Roots	48.33 ± 7.71 ^e^	52.97 ± 3.04 ^e^	40.97 ± 0.53 ^d^	41.04 ± 5.40 ^d^
Underground buds	29.91 ± 2.30 ^a^	36.95 ± 5.26 ^b,c,d^	32.36 ± 2.66 ^a,b,c^	30.28 ± 3.27 ^a,b^
Protein (g/100 g DM)				
Leaves	11.51 ± 0.12 ^h^	15.79 ± 0.36 ^j^	16.77 ± 0.09 ^k^	13.77 ± 0.14 ^i^
Stems	3.64 ± 0.09 ^c^	6.94 ± 0.00 ^d^	8.62 ± 0.08 ^e^	9.24 ± 0.21 ^f^
Roots	1.84 ± 0.00 ^a^	2.63 ± 0.03 ^b^	3.26 ± 0.04 ^c^	3.54 ± 0.05 ^c^
Underground buds	10.52 ± 0.01 ^g^	13.65 ± 0.27 ^i^	15.46 ± 0.14 ^j^	16.65 ± 0.05 ^k^
Total ash (g/100 g DM)				
Leaves	9.14 ± 0.02 ^g^	9.55 ± 0.04 ^h^	10.44 ± 0.08 ^i^	9.05 ± 0.40 ^g^
Stems	3.29 ± 0.22 ^a,b^	3.92 ± 0.15 ^c,d^	4.12 ± 0.03 ^d^	3.54 ± 0.05 ^a,b,c^
Roots	3.69 ± 0.09 ^b,c,d^	3.19 ± 0.08 ^a^	3.71 ± 0.05 ^b,c,d^	3.75 ± 0.26 ^c,d^
Underground buds	10.51 ± 0.02 ^i^	7.81 ± 0.34 ^e,f^	7.43 ± 0.07 ^e^	8.18 ± 0.07 ^f^
Dietary fiber (g/100 g DM)				
Leaves	57.96 ± 0.39 ^b^	52.14 ± 2.36 ^a^	57.39 ± 0.13 ^b^	59.60 ± 0.29 ^b^
Stems	73.87 ± 0.28 ^c^	72.55 ± 0.43 ^c^	77.39 ± 1.16 ^d^	74.57 ± 0.01 ^c,d^
Fat (g/100 g DM)				
Leaves	2.71 ± 0.10	2.74 ± 0.12	2.70 ± 0.14	2.63 ± 0.06
Stems	0.47 ± 0.03	0.45 ± 0.01	0.43 ± 0.03	0.41 ± 0.03
Metabolized carbohydrates (g/100 g DM)				
Leaves	18.69 ± 0.80 ^d,e^	19.78 ± 2.99 ^e^	12.70 ± 0.07 ^b^	14.95 ± 0.04 ^b,c^
Stems	18.75 ± 0.05 ^d,e^	16.15 ± 0.77 ^c,d^	9.43 ± 1.58 ^a^	12.24 ± 0.29 ^a,b^
Energy value (kcal/100 g DM)				
Leaves	261.1 ± 0.5 ^c^	271.2 ± 6.3 ^d^	256.9 ± 0.7 ^c^	257.7 ± 1.4 ^c^
Stems	241.5 ± 0.5 ^b^	241.5 ± 1.8 ^b^	230.9 ± 3.2 ^a^	238.8 ± 0.3 ^b^

Mean value ± standard deviation (*n* = 2); DM—dry matter; caloric value was calculated according to Commission Directive 2008/100/EC; a–k—values relating to the content of dry matter, individual nutrients, or the energy value, depending on the morphological part of the plant, and the additional nitrogen dose and type of fertilizer denoted by the same letter do not differ statistically significantly at *p* ≤ 0.05.

## Data Availability

The data presented in this study are available on request from the corresponding author.

## Supplementary Materials

The following supporting information can be downloaded at: https://www.mdpi.com/article/10.3390/molecules27217597/s1, Table S1: The content of individual polyphenols (g/100 g DM or mg/100 g DW) in various morphological parts of fragrant agrimony *Agrimonia procera* Wallr. from experimental cultivation; Table S2: Analytical parameters used for quantitative analysis.

## Sample Availability

Samples of the compounds are not available from the authors.
