# Supplementary material for: Nutritional and Polyphenolic Composition of Agrimonia procera Wallr. from Experimental Cultivation with Different Levels of Nitrogen Fertilization
Source: Molecules. 2022 Nov 5;27(21):7597. doi: 10.3390/molecules27217597 (PMC9654828; doi:10.3390/molecules27217597)
Supplement: Supplementary file 1 [file molecules-27-07597-s001.zip › Supporting Information S1.pdf]

Table S1. The content of individual polyphenols (g/100 g DM or mg/100 g DW) in various morphological parts of fragrant agrimony *Agrimonia procera* Wallr. from experimental cultivation

Morphological part of plant	Seed bed with a nitrogen content of 140 mg/dm^3			
	Additional nitrogen dose and type of fertilizer			
	0	25 mg/dm^3 ammonium nitrate	100 mg/dm^3 ammonium nitrate	100 mg/dm^3 urea
Agrimoniin (g/100g DW)				
Leaves	4.80 ± 0.20^{abc}	4.80 ± 0.33^{abc}	3.87 ± 0.26^{ab}	3.73 ± 0.27^{a}
Stems	4.73 ± 0.15^{abc}	5.66 ± 0.28^{c}	5.15 ± 0.46^{bc}	5.35 ± 0.68^{c}
Roots	7.94 ± 0.99^{d}	8.36 ± 0.60^{d}	7.78 ± 0.94^{d}	8.20 ± 0.62^{d}
Underground buds	16.02 ± 0.72^{g}	11.04 ± 2.63^{e}	10.07 ± 1.27^{e}	13.90 ± 2.63^{f}
Pedunculagin (mg/100g DW)				
Leaves	91.9 ± 6.4^{d}	77.0 ± 3.5^{c}	64.9 ± 4.3^{b}	61.7 ± 2.4^{b}
Stems	96.4 ± 12.1^{d}	120.0 ± 13.2^{f}	107.2 ± 10.7^{e}	108.0 ± 18.9^{e}
Roots	51.9 ± 5.4^{a}	52.6 ± 5.0^{a}	64.5 ± 5.4^{b}	66.8 ± 6.1^{b}
Underground buds	nd	nd	nd	nd
Ellagic acid (mg/100 g DW)				
Leaves	7.1 ± 0.9^{a}	4.7 ± 0.9^{a}	6.4 ± 1.1^{a}	6.2 ± 1.1^{a}
Stems	13.7 ± 1.0^{b}	14.9 ± 1.5^{b}	16.5 ± 3.2^{bc}	16.1 ± 2.0^{bc}
Roots	25.8 ± 4.7^{gh}	27.5 ± 1.7^{h}	24.5 ± 2.1^{fg}	27.3 ± 3.0^{gh}
Underground buds	22.3 ± 4.3^{ef}	20.1 ± 1.9^{de}	18.7 ± 2.7^{cd}	19.5 ± 3.5^{de}
Proathocyanidins (g/100 g DW)				
Leaves	2.89 ± 0.43^{efg}	2.63 ± 0.24^{def}	3.03 ± 0.35^{fg}	3.04 ± 0.28^{fg}
Stems	1.87 ± 0.19^{bc}	1.57 ± 0.25^{ab}	1.83 ± 0.25^{bc}	2.47 ± 0.40^{de}
Roots	3.27 ± 0.26^{g}	2.67 ± 0.26^{def}	2.73 ± 0.24^{ef}	2.16 ± 0.27^{cd}
Underground buds	1.62 ± 0.02^{ab}	1.23 ± 0.02^{a}	1.45 ± 0.17^{ab}	1.65 ± 0.06^{abc}
Free catechins (mg/100 g DW)				
Leaves	779.9 ± 21.5^{bcd}	682.6 ± 48.6^{bc}	828.7 ± 57.3^{cd}	935.9 ± 125.8^{de}
Stems	455.7 ± 41.8^{a}	409.2 ± 22.1^{a}	686.0 ± 140.9^{bc}	719.4 ± 17.9^{bc}
Roots	1304.2 ± 111.1^{g}	1099.7 ± 93.5^{f}	1178.2 ± 67.6^{fg}	1081.6 ± 111.2^{ef}
Underground buds	899.9 ± 223.3^{d}	655.3 ± 129.8^{b}	645.8 ± 104.2^{b}	621.5 ± 82.4^{b}
Quercetin arabinoglycoside (mg/100 g DW)				
Leaves	693.0 ± 51.9^{e}	537.8 ± 37.8^{cd}	536.3 ± 24.5^{cd}	522.3 ± 15.2^{cd}
Stems	92.1 ± 8.2^{a}	86.1 ± 7.9^{a}	82.0 ± 5.6^{a}	68.0 ± 6.6^{a}
Roots	nd	nd	nd	nd
Underground buds	551.1 ± 61.2^{d}	410.9 ± 67.9^{b}	413.1 ± 59.6^{b}	504.3 ± 70.8^{c}
Quercetin 3-O-rhamnosyloglucoside (mg/100 g DW)				
Leaves	63.5 ± 2.6^{d}	61.0 ± 3.8^{d}	55.3 ± 4.6^{c}	53.3 ± 2.8^{c}
Stems	nd	nd	nd	nd
Roots	nd	nd	nd	nd

Underground buds	25.2.±4.6^{a}	29.3±4.4^{a}	27.3±4.2^{a}	36.3±4.1^{b}
Quercet**in** 3-O-galactoside (mg/100g DW)				
Leaves	113.1±20.9^{f}	76.0±11.3^{d}	71.7±8.5^{cd}	69.0±8.9^{cd}
Stems	11.2±1.6^{b}	8.7±0.8^{a}	8.5±2.0^{a}	6.9±0.5^{a}
Roots	nd	nd	nd	nd
Underground buds	100.5±8.7^{e}	61.3±8.3^{b}	72.9±10.0^{cd}	109.7±15.9^{ef}
Keampferol 3-O-glucoside (mg/100 g DW)				
Leaves	74.2±6.7^{e}	56.8±3.4^{d}	55.8±6.0^{cd}	57.4±2.1^{d}
Stems	5.7±0.8^{a}	5.8±0.8^{a}	4.9±1.1^{a}	3.8±0.5^{a}
Roots	nd	nd	nd	nd
Underground buds	59.3±7.9^{d}	45.9±6.5^{b}	50.0±6.5^{bc}	62.5±13.1^{d}
Kaempferol-3-*O*-β-d-(6″-*E*-p-coumaroyl)-glucopyranoside* (mg/100 g DW)				
Leaves	41.1±6.8^{c}	36.2±3.3^{bc}	38.3±8.0^{c}	37.4±2.8^{bc}
Stems	7.8±0.8^{a}	7.9±1.1^{a}	7.2±2.5^{a}	5.0±0.9^{a}
Roots	nd	nd	nd	nd
Underground buds	29.5±5.2^{b}	34.4±7.8^{bc}	37.3±14.5^{bc}	30.8±5.8^{b}
Luteolin 7-O-glucuronide (mg/100 g DW)				
Leaves	190.7±14.0^{f}	155.0±12.1^{e}	134.8±7.2^{d}	131.4±10.3^{d}
Stems	7.4±0.8^{a}	8.5±0.6^{a}	7.6±1.5^{a}	6.1±0.6^{a}
Roots	nd	nd	nd	nd
Underground buds	98.3±9.6^{c}	68.1±11.2^{b}	70.5±8.5^{b}	92.1±24.1^{c}
Apigenin 7-O-glucuronide (mg/100g DW)				
Leaves	747.4±18.3^{f}	650.0±10.9^{e}	595.6±42.5^{d}	589.3±21.3^{d}
Stems	8.5±0.9^{a}	9.0±1.2^{a}	8.9±3.4^{a}	6.3±1.6^{a}
Roots	nd	nd	nd	nd
Underground buds	359.7±58.2^{c}	309.3±46.8^{b}	294.3±28.5^{b}	386.7±80.3^{c}

Mean value ± standard deviation (n=4); DM - dry matter; Kaempferol-3-*O*-β-d-(6″-*E*-p-coumaroyl)-glucopyranoside*- sum of kaempferol-3-*O*-β-d-(6″-*E*-p-coumaroyl)-glucopyranoside isomers; nd – not detected; a - h - values relating to the content of individual polyphenols depending on the morphological part of the plant and the additional nitrogen dose and type of fertilizer denoted by the same letter do not differ statistically significantly at $p \leq 0.05$.
