# Supplementary material for: Nutritional and Polyphenolic Composition of Agrimonia procera Wallr. from Experimental Cultivation with Different Levels of Nitrogen Fertilization
Source: Molecules. 2022 Nov 5;27(21):7597. doi: 10.3390/molecules27217597 (PMC9654828; doi:10.3390/molecules27217597)
Supplement: Supplementary file 1 [file molecules-27-07597-s001.zip › Supporting Information S2.pdf]

Table S2. Analytical parameters used for quantitative analysis

Substance	Linear range<br>mg/L	Calibration curves	R^2	LOD<br>mg/L	LOQ<br>mg/L
Pedunculagin	2.0-188.0	y = 10.839x + 3.180	0.9983	0.54	1.64
Agrimoniin	5.0 – 249.0	y = 18.190x – 4.580	0.9998	1.290	3.901
Ellagic acid	2.5 – 76.6	y = 16.736x – 25.347	0.9997	0.168	0.508
Quercetin 3-O-glucoside	4.1– 41.4	y = 19.976x – 39.975	0.9999	0.202	0.611
Quercetin 3-O-rhamnoglucoside	1.14 –11.4	y = 25.741x – 11.244	0.9997	0.219	0.663
Quercetin 3-O-galactoside	1.6 – 16.0	y = 20.33x – 18.862	0.9999	0.013	0.039
Kaempferol 3-O-glucoside	1.4 – 14.0	y = 16.234x – 11.244	0.9999	0.187	0.566
KpCG*	1.2 – 12.0	y = 10.282x – 5.400	0.9999	0.0419	0.127
Luteolin	1.23 – 12.30	y = 30.717x – 22.356	0.9999	0.019	0.058
Apigenin 7-O-glucoside	1.0 – 100.0	y = 17.314x – 26.702	0.9993	0.148	0.448

KpCG* - kaempferol-3-O-β-d-(6″-E-pcoumaroyl)-glucopyranoside (tiliroside).
